# Evaluation of the anti-malarial activity of crude extract and solvent fractions of the leaves of *Olea europaea* (*Oleaceae*) in mice

**DOI:** 10.1186/s12906-019-2567-8

**Published:** 2019-07-11

**Authors:** Desye Misganaw, Ephrem Engidawork, Teshome Nedi

**Affiliations:** 1 Pharmacology and Toxicology unit, Department of pharmacy, College of Medicine and Health Science, Wollo University, P.O. Box 1145, Dessie, Ethiopia; 2 Department of Pharmacology and Clinical Pharmacy, School of Pharmacy, Addis Ababa University, Addis Ababa, Ethiopia

**Keywords:** Antimalarial activity, *Plasmodium berghei*, *Olea europaea*, *Parasitemia*

## Abstract

**Background:**

Drug resistance poses a challenge to malaria control measures. This calls for discovery & development of new chemotherapeutic agents. This study therefore was initiated to investigate the antimalarial activity of *Olea europaea* against P*lasmodium berghei* infected mice and to further ascertain in which fraction (s) the constituents responsible for anti-malarial activity are concentrated.

**Methods:**

The leaves of *Olea europaea* were extracted by maceration using 80% methanol and the crude extract was then successively fractionated with solvents of differing polarity (chloroform, n-butanol and water). The anti-malarial activity of various doses of the extract and fractions (200, 400 and 600 mg/kg) was evaluated using chemo-suppressive, curative, and repository tests. Parameters, including parasitemia, rectal temperature, body weight, and packed cell volume were determined to establish the activity.

**Results:**

The acute oral toxicity test result revealed that the LD50 values of the extract and fractions were greater than 2000 mg/kg in mice. The crude extract significantly reduced parasitemia (*p* < 0.001) and prolonged survival time (*p* < 0.001), in a dose-dependent manner, in all tests, as compared to the negative control group. Higher parasitemia suppression (58%) was achieved with the larger dose (600 mg/kg) in the 4-day suppressive test, suggesting that the crude extract has largely a chemo-suppressive activity.

Parasitemia was significantly reduced (*p* < 0.001) by all fractions in all doses used when compared to the negative controls, with the rank order of n-butanol (51%) > chloroform>aqueous (21%) fractions. Larger (600 mg/kg) and middle (400 mg/kg) doses of the crude extract as well as the fractions ameliorated all the other parameters in a consistent manner, with the crude being more active than the fractions. Preliminary phytochemical analysis revealed the presence of secondary metabolites that were differentially distributed in the fractions.

**Conclusion:**

The findings collectively indicate that the plant is endowed with antimalarial activity, the activity being more in the crude extract than the fractions, owing to the presence of secondary metabolites that act independently or in synergy. The varying degree of antimalarial activity in the fractions suggests that non-polar and medium polar principles could be responsible for the observed activity.

## Background

Half of the world population is at risk of malaria [1], the most common, and severe parasitic mosquito-borne disease [2]. Moreover, malaria claims more lives in Africa than in any other continent, as more than 90% of worldwide malaria related deaths occur in this region, which makes it the second cause of death after HIV/AIDS [3].

No single antimalarial drug is effective against all liver and intra-erythrocytic forms of the parasite, which could co-exist in the same patient. As a result, complete elimination of the parasite infection may require more than one drug during treatment of an established infection [4]. Besides, efforts to develop an effective blood stage vaccine have not met with much success primarily because of antigenic diversity and a poor understanding of protective host immune responses [5, 6].

The genomic plasticity of the mosquito and the plasmodium parasite has added another dimension for the problem through increasing resistance to drugs, demanding an investment in research and development of newer agents for malaria control [6]. Hence, traditional medicinal plants could be considered as an alternative source of new drugs, as some antimalarial drugs (quinine, artemisinin) in use today were of plant origin [7].

*Olea europaea Linn* (family: Oleaceae) is an extensively used plant for different kind of ailments in traditional medicine of various countries. Its bark, fruits, leaves, wood, seeds, and oil are used in different forms, alone or sometimes in combination with other herbs [8–10]. In east Africa (including Ethiopia), root, bark and leaf extracts are used to treat malaria and other infections [11–13]. In vitro studies also reported that the dichloromethane/methanol (1:1) leaf extract of *O. europaea* possessed anti-plasmodial activity on *P. falciparum* with an IC50 of 12 μg/ml [14]. Moreover, extracts from different parts of the plant have been reported to exhibit various pharmacological activities, including antidiabetic [15], anticancer [16], antioxidant and antimicrobial [17], antihypertensive [18], antiviral [19] and anti-inflammatory [18].

Although ethno botanical and in vitro studies [11, 12, 14] showed that *O. europaea* has anti-malarial activity, no report is available in the literature whether the plant also possesses in vivo activity. This study therefore was initiated to investigate the anti-plasmodial activity of the plant in rodent model of malaria and as well as to further ascertain in which fraction (s) the constituents responsible for anti-malarial activity are concentrated so as to provide a clue about the nature of the phytochemical constituents responsible for its action.

## Methods

### Plant material

The leaves of *O. europaea* were collected from kirkos sub city, Addis Ababa, in October, 2015. Identification and authentication of the plant specimen was done by a taxonomist and a voucher specimen (DM 002/2015) was deposited at the National Herbarium, College of Natural and Computational Sciences, Addis Ababa University for future reference.

### Experimental animals and parasite

Male (for anti-malarial test) and female (for acute toxicity test) healthy Swiss albino mice, weighing 20–30 g and aged 6–8 weeks were used for the experiment. The animals were kept in plastic cages at room temperature and 12 h light and 12 h dark cycle, with free access to pellet food and water. The animals were acclimatized to laboratory condition for 1 week prior to the experiment. Chloroquine sensitive strain of *P. berghei* was used for the antimalarial tests. The parasite was maintained by passage of blood from infected to non-infected mice on weekly basis.

### Plant extraction and fractionation

The leaves were cleaned, dried under shade and then grounded into a coarse powder using mortar and pestle. About 600 g of the coarse plant material was weighed by sensitive digital weighing balance (METTLER TOLEDO, Switzerland) and extracted by cold maceration technique. The plant material was soaked in a flask containing about 1.5 L of 80% methanol and then placed on a shaker (Thermoforma, USA) at 120 rpm for 72 h at room temperature. The extract was filtered using Whatman grade No 1 filter paper and the marc was re-extracted twice by adding equal volume of fresh 80% methanol. The filtrates were combined and concentrated in a rotary evaporator (Buchi type TRE121, Switzerland) with temperature not exceeding 40 °C. The concentrated filtrate was then frozen in a freezer and dried in a lyophilizer (Wagtech Jouan Nordic DK-3450 Allerod, Denmark). About two-third of the crude 80% methanol extract (80ME) was then successively fractionated using chloroform, n-butanol, and water. To this effect, the crude extract was initially mixed with water. The suspension was then shaken in a separatory funnel by adding chloroform 3 times and the chloroform fraction was obtained. The aqueous residue was then shaken with n-butanol 3 times to obtain the n-butanol fraction. Thereafter, the n-butanol and chloroform fractions were concentrated in a rotary evaporator. The aqueous residue was also lyophilized to obtain the aqueous fraction. Finally, the crude extract and fractions were stored in a freezer (− 20 °C) until used for the experiment.

### Acute toxicity test

Acute oral toxicity test for crude and solvent fractions of the leaves of *O. europaea* was performed according to the organization for economic co-operation and development (OECD) guideline 425 [20]. Five female albino mice of 6–8 weeks were used for each test. All mice were fasted for 4 h before and 2 h after the administration of the 80ME and fractions. First, a sighting study was performed to determine the starting dose. For this, a single female mouse was given 2000 mg/kg of the 80ME and fractions as a single dose by oral gavage. Since no death was observed within 24 h, additional four mice were used, and administered the same dose of 80ME and fractions. The animals were observed continuously for 4 h with 30 min interval and then for 14 consecutive days with an interval of 24 h for the general signs and symptoms of toxicity, food and water intake and mortality.

### Animal grouping and dosing

In all models, animals were randomly divided into five groups (negative control, positive control and three test groups) comprising of six animals per group. Negative controls received vehicle (2% Tween 80 for 80ME, chloroform fraction and n-butanol fraction; distilled water for aqueous fraction; 10 ml/kg); and positive controls received chloroquine 25 mg/kg in all models. The test groups (group 3, 4 and 5) received different doses (200, 400 and 600 mg/kg respectively) of 80ME and fractions orally.

### Inoculation of mice

Parasitemia of the donor’s blood was first determined. These mice were then sacrificed by cervical dislocation, and blood was collected in a Petri dish with an anticoagulant (0.5% trisodium citrate) by severing the jugular vein. The blood was then diluted with physiological saline (0.9%) based on parasitemia of the donor mice and the red blood cells (RBC) count of normal mice in such a way that 1 ml blood contained 5 × 10^7^ infected erythrocytes. Each mouse then received 0.2 ml of diluted blood containing 1 × 10^7^ *P. berghei* infected erythrocytes by intraperitoneal (ip) route.

### Determination of anti-malarial activity

#### Four-day suppressive test

The Peter’s four-day suppressive test [21] was employed to test the chemo-suppressive activity of the plant against mice infected with chloroquine sensitive *P. berghei*. Thirty mice each for 80ME and solvent fractions testing were infected on the first day (day 0). Two-hour post-infection, the mice were randomly distributed into the five groups and treated as described in animal grouping and dosing section. Treatment was continued for additional three consecutive days at 24, 48 and 72 h post-infection (until day 3). On day 4 of the experiment (at 96 h post-infection), blood was collected from the tail of each mouse and thin smear were prepared on a microscope slides to determine parasitemia. In addition, mice weight, temperature and packed cell volume (PCV) were measured just before infection and at the end of the experiment. Afterwards, mice were followed for 28 (day 0-day 27) days so as to determine the mean survival time (MST) for each group.

#### Curative test

Rane’s test, which evaluates the curative potential of extracts, was carried out according to the method described by Ryley and Peters [22].Thirty mice were infected on the first day (Day 0) prior to administration of 80ME. After 72 h (day 3), the animals were randomly assigned into five groups with six mice in each group and treated with their respective doses as described earlier in animal grouping and dosing section. Treatment was continued for further 3 days (i.e. 96, 120, 144 h post-infection). Parasitemia level was recorded daily throughout the experiment beginning from day 3. Other parameters also determined as described in the chemo-suppressive test section.

#### Prophylactic test

Evaluation of the prophylactic potential of the extract was done according to the method described by Peters [23]. For 80ME, 30 mice were randomly distributed into five groups of six mice each and treated as described earlier in animal grouping and dosing section. Twenty-four hour after a single treatment (day 0), all the groups were infected with inoculum of 1 × 10^7^ *P. berghei* infected erythrocytes. Blood smears were drawn 72 h post-infection (day 3) from each mouse to determine parasitemia level. Other parameters also determined as described in the chemo-suppressive test section.

#### Determination of parasitemia and survival time

A thin smear of blood from each mouse was applied on a different microscope slide. The smear was fixed with absolute methanol and stained with 10% Geimsa stain for 15 min. The slides were then taken out, washed with gentle passage of tap water and dried at room temperature. With little drop of oil immersion, the number of parasite-infected red blood cells (pRBC) were counted using a light microscope (MB23 0 T, China) with an objective lens magnification power of 100x. The parasitemia was determined by counting a minimum of three fields per slide. Percent parasitemia and percent inhibition were calculated using the modified Peters and Robinson formula [24]:

$$ \% Parasitemia=\frac{Number\ of\ parasitized\  RBC}{\  Total\ number\ of\  RBC\  count}\times 100 $$

$$ \% Suppression=\frac{\% Parasitemia\ in\ negative\ control-\% Parasitemia\ in\ study\ group\ }{\% Parasitemia\ in\ negative\ control} \times 100 $$

Finally, the animals were followed and their MST was determined using the formula indicated below as described elsewhere [25].

$$ MST=\frac{Total\ number\ of\ days\ mice\ survived}{Total\ number\ of\ mice} $$

#### Determination of packed cell volume, rectal temperature and body weight

Blood was collected from tail of each mouse in heparinized microhaematocrit capillary tubes to 3/4th of their original height and sealed at their dry end with sealing clay. The tubes were then placed in a microhematocrit centrifuge (Hettichhaematokrit, Germany) with the sealed ends outwards. The blood was centrifuged at 12,000 rpm for 5 min. PCV was determined using the following relation [26].

$$ PCV=\frac{Volume\ of\ erythrocytes\ in\ a\  given\ volume\ of\ blood}{Total\ blood\ volume}\times 100 $$

Each mouse in a group was weighed using sensitive digital weighing balance and rectal temperature was measured using digital rectal thermometer. The percentage changes of their mean values that occurred before and after treatment were then calculated.

#### Preliminary phytochemical screening

The qualitative phytochemical investigations of 80ME and fractions (chloroform, n-butanol and aqueous) were carried out using standard tests [27, 28].

#### Data analysis

Data was analyzed using windows SPSS version 20.0. Results obtained from the study are expressed as mean ± standard error of the mean (SEM). One-way ANOVA followed by Tukey’s post hoc test for multiple comparisons was used to compare results among groups. In addition, two way repeated measures ANOVA was also used to analyze the development of parasitemia across days of treatment in Rane’s test. The result was considered statistically significant at 95% confidence level when *p*-value was < 0.05.

## Results

### Acute oral toxicity test

The acute oral toxicity test of 80ME and solvent fractions of leaves of *O. europaea* indicated that neither the 80ME nor the solvent fractions caused gross behavioral changes and mortality within 24 h as well as in the following 14 days, indicating that the LD50 values of the extract and fractions were greater than 2000 mg/kg in mice.

### Effect of 80% methanol extract in the 4-day suppressive test

The results of the 4-day suppressive test indicated that the 80ME reduced (*p* < 0.001 in all cases) parasitemia in a dose-dependent manner compared to negative control, with percentage suppression of about 50, 55, 58 for 200 mg/kg, 400 mg/kg and 600 mg/kg doses, respectively (Table 1). Likewise, MST was significantly prolonged (*p* < 0.001) by all doses of the extract. Nevertheless, both effects observed were significantly lower (*p* < 0.001) than the standard drug.

**Table 1:** Parasitemia and survival time of infected mice treated with 80% methanol extract of the leaves of *Olea europaea* in the 4-day suppressive test

Groups	% Parasitemia	% Suppression	Survival time (days)
2% TW80	37.96 ± 0.63	–	7.16 ± 0.17
CQ 25 mg/kg	0.00 ± 0.00	100.00^a3^	28.00 ± 0.00^a3^
200 mg/kg	19.07 ± 0.34	49.75^a3b3d3e3^	11.33 ± 0.21^a3b3d2e3^
400 mg/kg	17.15 ± 0.12	54.82^a3b3e3^	13.00 ± 0.36^a3b3e3^
600 mg/kg	16.02 ± 0.06	57.78^a3b3^	15.16 ± 0.30^a3b3^

Data are expressed as mean ± SEM (Standard Error of the Mean); *n* = 6; a, compared to negative control; b, to CQ25 mg/kg; c, to 200 mg/kg; d, to 400 mg/kg; e, to 600 mg/kg; 1, *p* < 0.05; 2, *p* < 0.01; 3, *p* < 0.001; 2% TW80, 2% Tween80; *CQ* chloroquine

PCV determination revealed that the 80ME exhibited a statistically significant effect (*p* < 0.001) in the circumvention of PCV decline at all doses compared to the negative control even if the effect was still lower than the standard drug (*p* < 0.001 in all cases) (Table 2).

**Table 2:** Packed cell volume, rectal temperature and body weight of infected mice treated with 80% methanol extract of the leaves of *Olea europaea* in the 4-day suppressive test

Groups	Packed cell volume			Rectal temperature			Body weight		
	D0	D4	% change	D0	D4	% change	D0	D4	% change
2% TW80	61.81 ± 1.16	56.19 ± 1.09	−9.09	36.74 ± 0.06	34.8 ± 0.18	−5.28	28.25 ± 0.41	26.93 ± 0.41	−4.66
CQ25 mg/kg	61.52 ± 1.13	61.20 ± 1.12	−0.52^a3^	35.52 ± 0.27	35.51 ± 0.20	−0.03^a3^	28.93 ± 0.34	29.26 ± 0.37	1.13^a3^
200 mg/kg	59.61 ± 0.68	56.60 ± 0.77	−5.05^a3b3d3e3^	36.23 ± 0.20	34.96 ± 0.17	−3.50^a2b3e2^	28.95 ± 0.32	27.79 ± 0.35	−4.00^b3^
400 mg/kg	62.08 ± 0.77	60.15 ± 0.73	−3.10^a3b3^	36.46 ± 0.17	35.67 ± 0.22	−2.16^a3b2^	29.03 ± 0.49	28.05 ± 0.47	−3.39^a2b3^
600 mg/kg	62.05 ± 0.65	60.51 ± 0.67	−2.49^a3b3^	36.00 ± 0.18	35.56 ± 0.16	−1.22^a3^	28.02 ± 0.74	27.16 ± 0.79	−3.06^a2b3^

Data are expressed as mean ± SEM; n = 6; a, compared to negative control; b, to CQ25 mg/kg; c, to 200 mg/kg; d, to 400 mg/kg; e, to 600 mg/kg; 1, *p* < 0.05; 2, *p* < 0.01; 3, *p* < 0.001; 2% TW80, 2% Tween80; *CQ* chloroquine, *D0* pre-treatment value on day 0, *D4* post- treatment value on day 4

As regards to rectal temperature, all doses prevented the reduction of rectal temperature due to infection by *P. berghei* significantly (*p* < 0.01 for 200 mg/kg, *p* < 0.001 for 400 mg/kg and 600 mg/kg) as compared to negative control group. Still, the higher dose of the extract was the only dose displaying statistically comparable effect in temperature stabilization compared to the standard drug (Table 2).

With respect to body weight, the middle and higher doses attenuated body weight reduction significantly (*p* < 0.01) in comparison with the negative control. However, the effect was significantly lower (*p* < 0.001) than the standard and no detectable changes were also observed among the various doses of the crude extract.

### Effect of 80% methanol extract in the Rane’s test

**Fig. 1:**
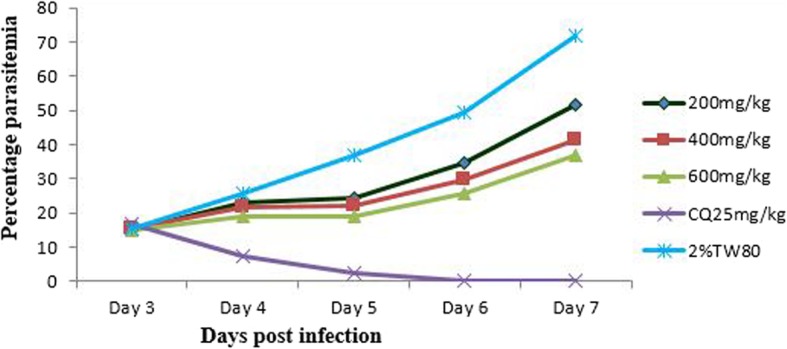
Parasitemia development over the course of treatment with 80% methanol extract of the leaves of *Olea europaea* in Rane’s model

Although there was a gradual escalation of parasitemia throughout the course of treatment in all the groups, the increment in percentage parasitemia of negative control group was relatively higher than that of the extract treated groups (Fig. 1). Indeed, two way repeated measures ANOVA analysis of parasitemia showed significant difference (*p* < 0.001) in parasite development across the course of treatment. Analysis of percentage suppression revealed that extract treated groups (all the three doses) had significant curative effect (*p* < 0.001) as compared to negative control group, although the efficacy was lower (*p* < 0.001) than the positive control group. Moreover, significantly different effect (*p* < 0.001 in all cases) was also detected among the doses, probably reflecting a dose-dependent effect of the extract (Table 3).

**Table 3:** Parasitemia and survival time of infected mice treated with 80% methanol extract of the leaves of *Olea europaea* in Rane’s test

Groups	% Parasitemia	% Suppression	Survival time (days)
2% TW80	71.67 ± 0.79	–	7.66 ± 0.21
CQ25 mg/kg	0.00 ± 0.00	100.00^a3^	28.00 ± 0.00^a3^
200 mg/kg	51.55 ± 0.71	28.08^a3b3d3e3^	11.16 ± 0.30^a3b3d1e3^
400 mg/kg	41.23 ± 0.64	42.77^a3b3e3^	12.50 ± 0.34^a3b3e2^
600 mg/kg	36.73 ± 0.35	48.75^a3b3^	14.17 ± 0.31^a3b3^

Data are expressed as mean ± SEM; n = 6; a, compared to negative control; b, to CQ25 mg/kg; c, to 200 mg/kg; d, to 400 mg/kg; e, to 600 mg/kg; 1, *p* < 0.05; 2, *p* < 0.01; 3, *p* < 0.001; 2% TW80, 2% Tween80; *CQ* chloroquine

The result of MST indicated that all the three test doses prolonged their respective groups survival time significantly (*p* < 0.001 in all cases) as compared to the negative control group. However, once again, the standard drug was more effective (*p* < 0.001) than the test doses.

A significant dose dependent effect (*p* < 0.001) was observed in the prevention of PCV reduction in all test doses, in a dose-dependent manner, when compared to the negative control, even though their effect was less (*p* < 0.001) than the standard drug (Table 4).

**Table 4:** Packed cell volume, rectal temperature and body weight of infected mice treated with 80% methanol extract of the leaves of *Olea europaea* in Rane’s test

Groups	Packed cell volume			Rectal temperature			Body weight		
	D3	D7	% change	D3	D7	% change	D3	D7	% change
2% TW80	51.86 ± 0.53	43.20 ± 0.36	−16.69	35.36 ± 0.17	33.51 ± 0.13	−5.25	28.43 ± 0.72	25.70 ± 0.74	−9.65
CQ25 mg/kg	50.31 ± 0.42	51.84 ± 0.35	3.05^a3^	35.18 ± 0.11	35.47 ± 0.13	0.83^a3^	28.63 ± 0.61	29.07 ± 0.63	1.51^a3^
200 mg/kg	51.93 ± 0.59	46.62 ± 0.63	−10.23^a3b3d2e3^	35.31 ± 0.25	33.71 ± 0.24	−4.52^a1b3d1e3^	27.00 ± 0.60	24.71 ± 0.64	−8.50^b3^
400 mg/kg	51.84 ± 0.68	47.86 ± 0.70	−7.68^a3b3^	35.36 ± 0.09	34.05 ± 0.13	−3.72^a3b3^	28.22 ± 0.49	26.04 ± 0.46	−7.71^a2b3^
600 mg/kg	51.78 ± 0.43	48.26 ± 0.53	−6.81^a3b3^	35.36 ± 0.07	34.23 ± 0.06	−3.18^a3b3^	27.63 ± 0.49	25.59 ± 0.50	−3.39^a2b3^

Data are expressed as mean ± SEM; n = 6; a, compared to negative control; b, to CQ25 mg/kg; c, to 200 mg/kg; d, to 400 mg/kg; e, to 600 mg/kg; 1, *p* < 0.05; 2, *p* < 0.01; 3, *p* < 0.001; 2% TW80, 2% Tween80; *CQ* chloroquine, *D3* pre-treatment value on day 3, *D4* post- treatment value on day 7

On the other parameter of the study, rectal temperature, the extract dose-dependently brought about a significant (*p* < 0.001 for middle and higher, and *p* < 0.05 for lower doses) prevention in temperature drop compared to the negative controls, albeit the effect was lower than the standard (Table 4).

Attenuation of body weight loss was achieved with the middle and higher doses (*p* < 0.001), but the lower dose failed to bring a statistical effect. Although no detectable changes were observed among the doses, all had a significantly lower effect (*p* < 0.001) than the standard drug.

### Effect of 80% methanol extract in the prophylactic test

Despite the overall less prophylactic effect compared to the positive control group, all doses produced a dose-related significant changes (*p* < 0.001) in parasitemia suppression as compared to the negative control group (Table 5).

**Table 5:** Parasitemia and survival time of infected mice treated with 80% methanol extract of the leaves of *Olea europaea* in prophylactic test

Groups	% Parasitemia	% Suppression	Survival time (days)
2% TW80	20.95 ± 0.27	–	6.33 ± 0.21
CQ25 mg/kg	4.7 ± 0.17	77.53^a3^	12.50 ± 0.22^a3^
200 mg/kg	16.87 ± 0.23	19.32^a3b3d3e3^	7.33 ± 0.21^a1b3d2e3^
400 mg/kg	15.17 ± 0.23	27.47^a3b3e1^	8.67 ± 0.21^a3b3^
600 mg/kg	14.08 ± 0.11	32.69^a3b3^	9.17 ± 0.17^a3b3^

Data are expressed as mean ± SEM; n = 6; a, compared to negative control; b, to CQ25 mg/kg; c, to 200 mg/kg; d, to 400 mg/kg; e, to 600 mg/kg; 1, *p* < 0.05; 2, *p* < 0.01; 3, *p* < 0.001; 2% TW80, 2% Tween80; *CQ* chloroquine

More or less a significant pattern was emerged in survival time changes as the parasitemia. All treatment groups prolonged survival time significantly compared to the negative controls, with the standard exhibiting a highly significant prolongation (*p* < 0.001) than the extract (Table 5).

The three doses significantly attenuated the reduction in PCV caused by parasite infection (*p* < 0.001) as compared to negative control in a dose dependent manner. However, the effect of chloroquine treated group on PCV was still higher (*p* < 0.001) than extract treated groups (Table 6). As to rectal temperature, the middle and the higher doses exhibited statistically significant (*p* < 0.001) preventive effect in rectal temperature reduction as compared to negative control mice (Table 6).

**Table 6:** Packed cell volume, rectal temperature and body weight of infected mice treated with 80% methanol extract of the leaves of *Olea europaea* in prophylactic test

Groups	Packed cell volume			Rectal temperature			Body weight		
	D 0	D3	% change	D0	D3	% change	D0	D3	% change
2% TW80	58.56 ± 0.38	56.47 ± 0.38	−3.57	36.10 ± 0.14	35.20 ± 0.14	−2.49	28.35 ± 0.40	27.65 ± 0.39	−2.45
CQ25 mg/kg	58.70 ± 0.65	58.59 ± 0.67	−0.19^a3^	35.81 ± 0.22	35.74 ± 0.20	0.22^a3^	28.77 ± 0.38	28.87 ± 0.38	0.36^a3^
200 mg/kg	58.65 ± 0.49	57.3 ± 0.48	−2.30^a3b3d1e2^	36.06 ± 0.12	35.30 ± 0.11	−2.08^b3d2e2^	28.76 ± 0.38	28.09 ± 0.38	−2.32^b3^
400 mg/kg	59.10 ± 0.49	57.91 ± 0.47	−2.01^a3b3^	36.40 ± 0.14	35.86 ± 0.13	−1.50^a3b3^	29.04 ± 0.34	28.40 ± 0.35	−2.20^a1b3^
600 mg/kg	58.70 ± 0.66	57.56 ± 0.67	−1.91^a3b3^	36.07 ± 0.13	35.71 ± 0.13	−1.00^a3b3^	28.86 ± 0.55	28.24 ± 0.53	−2.13^a2b3^

Data are expressed as mean ± SEM; n = 6; a, compared to negative control; b, to CQ25 mg/kg; c, to 200 mg/kg; d, to 400 mg/kg; e, to 600 mg/kg; 1, *p* < 0.05; 2, *p* < 0.01; 3, *p* < 0.001; 2% TW80, 2% Tween80; *CQ* chloroquine, *D0* pre-inoculation value on day 0, *D3* post- treatment value on day 3 after inoculation

Relating to body weight, there were no significant difference among the three doses. However, the middle and higher test doses of the extract alleviated the weight reduction in comparison with the negative control group (Table 6).

### Effect of solvent fractions on the 4-day suppressive test

Parasitemia was significantly reduced (*p* < 0.001) by all fractions in all doses used when compared to negative controls, with the rank order of n-butanol>chloroform>aqueous fractions. However, the effect was still significantly lower (*p* < 0.001) when compared to the standard drug (Table 7). While only the higher dose of the aqueous fraction produced significant effect with its lower dose, both the middle and higher doses of n-butanol and chloroform fractions showed statistically significant effect in comparison with their lower test doses (Table 7).

**Table 7:** Parasitemia and survival time of infected mice treated with solvent fractions of the leaves of *Olea europaea* in the 4-day suppressive test

Groups	% Parasitemia	% Suppression	Survival time (days)
2% TW80	37.84 ± 0.51	–	7.33 ± 0.21
CQ25 mg/kg	0.00 ± 0.00	100.00^a3^	28.00 ± 0.00^a3^
200 mg/kg CF	25.92 ± 0.25	31.52^a3b3d2e3^	10.17 ± 0.31^a3b3e1^
400 mg/kg CF	24.17 ± 0.24	36.12^a3b3^	10.83 ± 0.33^a3b3^
600 mg/kg CF	23.45 ± 0.13	38.03^a3b3^	11.17 ± 0.17^a3b3^
2% TW80	38.01 ± 0.61	–	7.17 ± .017
CQ25 mg/kg	0.00 ± 0.00	100.00^a3^	28.00 ± 0.00^a3^
200 mg/kg BF	22.55 ± 0.28	40.68^a3b3d2e3^	11.33 ± 0.33^a3b3d1e3^
400 mg/kg BF	20.44 ± 0.45	46.23^a3b3e1^	12.67 ± 0.33^a3b3e1^
600 mg/kg BF	18.77 ± 0.22	50.63^a3b3^	13.83 ± 0.31^a3b3^
2% TW80	37.86 ± 0.51	–	7.17 ± 0.17
CQ25 mg/kg	0.00 ± 0.00	100.00^a3^	28.00 ± 0.00^a3^
200 mg/kg AF	31.25 ± 0.78	17.46^a3b3e1^	8.83 ± 0.17^a3b3e1^
400 mg/kg AF	30.34 ± 0.26	19.86^a3b3^	9.50 ± 0.22^a3b3^
600 mg/kg AF	30.03 ± 0.27	20.67^a3b3^	9.83 ± 0.17^a3b3^

Data are expressed as mean ± SEM; n = 6; CF, chloroform fraction; BF, butanol fraction; AF, aqueous fraction; a, compared to negative control; b, to CQ25 mg/kg; c, to 200 mg/kg; d, to 400 mg/kg; e, to 600 mg/kg; 1, *p* < 0.05; 2, *p* < 0.01; 3, *p* < 0.001; 2% TW80, 2% Tween80; *CQ* chloroquine

The MST of all the fractions exhibited significant effect (*p* < 0.001) with their respective negative control groups even if the effect of the standard drug was more profound (*p* < 0.001) than all doses of the three fractions. Comparison among the study doses indicated that the higher dose of all the fractions had significant effect (*p* < 0.001) when compared to their respective lower dose treated groups. At the same time, the middle dose group of n-butanol fraction had significant effect (*p* < 0.05) with the lower dose treated group, while that of aqueous and chloroform fractions didn’t (Table 7).

The 4-day suppressive test on PCV showed that all the test doses of the three fractions produced significant protective effect (*p* < 0.001), with the same rank order as that of parasitemia. However, the positive control groups of all the fractions were more effective (*p* < 0.001) than their respective test and negative control groups. While both the middle and higher test doses of n-butanol fraction displayed significant effect in comparison with its lower dose, only the higher dose treated group of chloroform fraction showed significant difference effect with its lower dose treated group. Nonetheless, comparison among the three doses of aqueous fraction didn’t show statistically significant different effect (Table 8).

**Table 8:** Packed cell volume, rectal temperature and body weight of infected mice treated with solvent fractions of the leaves of *Olea europaea* in the 4-day suppressive test

Groups	Packed cell volume			Rectal temperature			Body weight		
	D 0	D4	% change	D0	D4	% change	D0	D4	% change
2% TW80	59.51 ± 0.41	54.24 ± 0.51	−8.86	36.71 ± 0.08	34.83 ± 0.17	−5.12	28.57 ± 0.26	27.26 ± 0.22	−4.58
CQ25 mg/kg	60.32 ± 0.52	60.03 ± 0.52	−0.48^a3^	35.67 ± 0.28	35.68 ± 0.24	0.03^a3^	28.27 ± 0.21	28.61 ± 0.24	1.20^a3^
200 mg/kg CF	59.7 ± 0.70	56.11 ± 0.68	−5.85^a3b3e1^	36.50 ± 0.22	35.18 ± 0.21	−3.61^a1b3^	28.33 ± 0.15	27.15 ± 0.15	−4.17^b3^
400 mg/kg CF	60.18 ± 0.49	56.99 ± 0.44	−5.14^a3b3^	36.39 ± 0.17	35.53 ± 0.19	−3.46^a2b3^	28.16 ± 0.25	27.17 ± 0.28	−3.52^a1b3^
600 mg/kg CF	60.88 ± 0.34	57.99 ± 0.40	−4.75^a3b3^	35.92 ± 0.18	35.30 ± 0.16	−3.12^a2b3^	28.35 ± 0.73	27.4 ± 70	−3.35^a2b3^
2% TW80	59.51 ± 0.41	54.21 ± 0.51	−8.91	36.71 ± 0.08	34.78 ± 0.17	−5.26	28.59 ± 0.26	27.26 ± 0.37	−4.65
CQ25 mg/kg	60.32 ± 0.52	60.03 ± 0.51	−0.48^a3^	35.68 ± 0.29	35.68 ± 0.24	0.00^a3^	28.29 ± 0.22	28.61 ± 0.24	1.13^a3^
200 mg/kg BF	59.63 ± 0.70	56.47 ± 0.75	−5.30^a3b3d1e2^	36.55 ± 0.22	35.25 ± 0.20	−3.52 ^a1b3e2^	28.20 ± 0.2	27.04 ± 0.18	−4.11^b3^
400 mg/kg BF	60.23 ± 0.49	57.73 ± 0.44	−4.15^a3b3^	36.44 ± 0.17	35.62 ± 0.20	−2.25^a3b3^	28.16 ± 0.32	27.21 ± 0.35	−3.37^a1b3^
600 mg/kg BF	60.93 ± 0.34	58.69 ± 0.40	−3.68^a3b3^	35.97 ± 0.18	35.5 ± 0.16	−1.31^a3^	27.85 ± 0.73	26.99 ± 0.71	−3.09^a2b3^
2% TW80	59.44 ± 0.41	54.22 ± 0.51	−8.78	36.69 ± 0.10	34.78 ± 0.14	−5.21	28.59 ± 0.26	27.26 ± 0.22	−4.65
CQ25 mg/kg	60.30 ± 0.51	60.03 ± 0.51	-.45^a3^	35.68 ± 0.28	35.67 ± 0.24	−0.03^a3^	28.29 ± 0.22	28.61 ± 0.24	1.13^a3^
200 mg/kg AF	59.75 ± 0.70	55.18 ± 0.68	−5.98^a3b3^	36.57 ± 0.22	35.14 ± 0.20	−3.90^a1b3^	28.36 ± 0.15	27.16 ± 0.16	−4.33^b3^
400 mg/kg AF	60.22 ± 0.49	55.87 ± 0.43	−5.57^a3b3^	36.43 ± 0.17	35.23 ± 0.19	−3.61^a2b3^	28.2 ± 0.25	27.01 ± 0.28	−4.22 ^b3^
600 mg/kg AF	60.86 ± 0.35	56.69 ± 0.40	−5.22^a3b3^	36.02 ± 0.18	35.10 ± 0.16	−3.43^a2b3^	28.42 ± 0.73	27.31 ± 0.69	−3.91 ^b3^

Data are expressed as mean ± SEM; n = 6; CF, chloroform fraction; BF, butanol fraction; AF, aqueous fraction; a, compared to negative control; b, to CQ25mg/kg; c, to 200 mg/kg; d, to 400 mg/kg; e, to 600 mg/kg; 1, *p* < 0.05; 2, *p* < 0.01; 3, *p* < 0.001; 2% TW80, 2% Tween80; *CQ* chloroquine, *D0* pre -treatment value on day 0, *D4* post- treatment value on day 4

All the three fractions showed significant effect on the ablation of rectal temperature drop as compared to their negative control groups with the effect of n-butanol fraction being the highest followed by chloroform fraction. The higher dose of n-butanol fraction was the only dose among the fractions to show statistically comparable effect as compared to the standard drug (*p* > 0.05). Additionally, analysis among the test doses of the fractions indicated that only the higher dose of n-butanol fraction brought about significant effect (*p* < 0.05) as compared to its lower dose (Table 8).

As to the parameter of body weight, only the middle and higher doses of n-butanol and chloroform fractions indicated statistically significance effect as compared to their negative control groups as presented in Table 8. While the positive control showed significance effect (*p* < 0.001) as compared to negative control as well as to all the test doses of the three fractions, statistical significance effect wasn’t noticed among the three test doses of all the fractions.

### Preliminary phytochemical screening

The preliminary phytochemical screening of 80ME revealed the presence of all tested constituents except saponins. Polyphenols, terpenoids, alakloids and flavonoids were detected in both chloroform and n-butanol fractions whereas polyphenols, tannins and glycosides detected in aqueous fraction (Table 9).

**Table 9:** Preliminary phytochemical screening of 80% methanol extract and solvent fractions of the leaves of *Olea europaea*

Metabolites	80% Methanol extract	Solvent fractions		
		Chloroform fraction	N-butanol fraction	Aqueous fraction
Polyphenols	+	+	+	+
Terpenoids	+	+	+	–
Flavonoids	+	+	+	–
Steroids	+	+	+	–
Alkaloids	+	+	+	–
Glycosides	+	–	+	+
Tannins	–	–	–	+
Saponins	–	–	–	–

**+** = presence, − **=** absence

## Discussion

The 80ME of the plant was investigated for its antimalarial effect using three models. Peter’s 4-day suppressive test was used to evaluate schizontocidal activity, during early infection while the Rane’s test was used to study curative ability during established infection and the repository test was used to study the prophylactic activity of the plant. In all methods, determinations of percent inhibition of parasitemia and survival time were the most reliable parameters [29] and compounds are considered active when reduction in parasitemia is ≥30% [30]. Accordingly, all the test doses of the crude extract as well as the chloroform and n-butanol fractions presumed to be active in the four-day suppressive test.

In the 4-day suppressive activity, the crude extract inhibited the level of parasitemia in a dose dependent manner, confirming the potential schizontocidal activity of the plant extract in early infection whereby the primary attack due to malaria can be prevented or mitigated [21]. Along with it, all the three doses of the extract improved survival time of infected mice, indicating its parasite suppressive activity and thereby reduction of the overall pathogenic effect of the parasite on test groups [31].

In the second model (Rane’s test), there was significant parasitemia suppression in all the doses used during the course of treatment, suggesting the curative potential of the extract. Justifying the probable rapid action of the extract [32], the relative suppression of parasitemia in extract treated mice started after the first dose as compared to the negative control group (Fig. 1). The overall lower curative than suppressive effect could possibly be due to short duration of action of the constituent(s) to cover the exponentially growing parasites in established infection [31]. This could be supported by the observation that oleuropein and its metabolite (hydroxytyrosol), chief constituents of the leaf of *O. europaea*, are rapidly absorbed after oral administration with a maximum plasma concentration occurring 2 h after administration and rapidly distributed and excreted in urine [33, 34]. This is in agreement with other studies where crude extracts had less effect on established infection than early infection [35, 36].

In the prophylactic test, the extract produced the lowest percentage suppression of parasitemia as compared to its effect in the 4-day suppressive and curative tests; however, all the doses of the extract demonstrated significant suppressive effect on the level of parasitemia compared to negative control group. The lower chemo-suppressive effect of the crude extract in prophylactic test might have arisen from rapid metabolism that inactivates the active component of the extract responsible for antimalarial activity [37]. Another possibility would be that the extract might have acted through metabolic activation of the immune system [38] and hence parasite clearance could not be total. This finding is in agreement with other studies in which the chemo-suppressive effect of prophylactic test is lower than that of 4-day suppressive and curative effects [39].

To further concentrate active principle responsible for the antimalarial activity, the powder of the 80ME was successively fractionated by solvents of differing polarity and their antimalarial activity were evaluated [29].

The results revealed that the n-butanol fraction showed the highest parasitemia suppression followed by the chloroform fraction and then the aqueous fraction, with maximum percentage suppression about 51, 39 and 21 at their higher dose, respectively. Likewise, survival time was better prolonged in n-butanol and chloroform fractions than aqueous fraction which could be ascribed to the relative higher parasitemia clearance (reduced parasite burden) observed for these fractions [40]. Besides, the highest and least chemo-suppressive effects of n-butanol and aqueous fractions, respectively, are in agreement with reports of other studies [26, 41]. This indicates the difference in the type and concentration of the bioactive secondary metabolites in the fractions, the most active subgroups being localized in the n-butanol and chloroform fractions (Table 9).

The 4-day chemo-suppressive effect of the 80ME exhibited more suppression than the fractions. Besides, the crude extract treated mice displayed improved survival time than mice treated with the fractions. The reduction in activity of the crude extract upon fractionation could be explained by the loss of additive or synergistic action among the chemical compounds in the extract and/or less concentration of bioactive compounds in the fractions [26]. This finding is in agreement with other studies in which the fractions reported to show less in vivo antimalarial activity than the crude extracts [26, 42].

A potent antimalarial is expected to ameliorate anemia, prevent body weight loss, and stabilize temperature in infected mice with parasite [43]. PCV was measured to determine the effectiveness of 80ME and solvent fractions of the leaf of *O. europaea* in preventing malaria-induced hemolysis alongside its antimalarial activity. Accordingly, the crude extract (in all the three models) as well as the three fractions of the plant prevented the reduction in PCV of parasite infected mice when compared with their respective negative control groups, implying that the extract could avert anemia due to malaria infection which might be due to destruction (clearance) and/or sequestration of infected erythrocytes [43].

Although the aqueous fraction displayed parasitemia reduction of < 30%, it showed significant protective effect on PCV. This could be probably due to the presence of phenols and other metabolites which have anti-oxidant and membrane protecting effects [17, 44]. Hydroxyl groups of phenolic compounds displays acidic characteristics, which makes them excellent antioxidants due to the electron donating activity [45]. The result of PCV, protective effect, in this study is concordant with the findings of Wannang et al [46] and Saba et al [47], however it is not in agreement with others [25]. The inconsistency may be due to the absence of detectable concentration of phytodetergents like saponins which destroy cell membrane by prompting cholesterol liberation that results erythrocyte hemolysis and PCV reduction [48].

The ability of the plant extract to prevent PCV reduction may be due to clearance of parasites from infected erythrocytes before hemolysis and/or by improving erythropoiesis [49] through differentiation inducing effects or generation of RBCs of olive leaf compounds such as apigenin 7-glucoside and luteolin 7-glucoside on hematopoietic stem cells [50]. Apart from this, decreased invasion and impaired intra-erythrocytic development of the parasites could be also responsible for the protective effect of RBC abnormalities [51]. Moreover, malaria infection activates the immune system and thereby causing the release of free radicals and reactive oxygen species that results in degradation of haemoglobin and development of anemia [52, 53]. Yet, the antioxidant properties of *O. europaea* leaf extract [17] especially polyphenolic compounds may protect RBCs from oxidative stress and help prolong the survival of both normal and infected RBCs during malaria infection.

In rodents, infection with parasites (increased parasitemia) results in decreased metabolic rates and severe hypothermia that could lead to death [54]. However, the extract showed the temperature stabilizing effect in all cases of 80ME and the solvent fraction test groups, with the effect of n-butanol being the highest among the fractions. This may, in addition to parasite suppression, probably indicate that the extract controlled the immune system of infected mice as well as adjusted some pathological processes and offset the reduction in metabolic rate that caused drop in internal body temperature. The effect seen in the aqueous fraction, despite the low parasitemia suppression, indicates the presence of secondary metabolites that could stabilize body temperature in the presence of infection (parasite) [55]. Moreover, ethno-botanical studies described that the plant is helpful in regulating body temperature in humans [56]. Therefore, the plant has a promising effect in stabilization of body temperature for malaria infection together with its antimalarial effect.

The decrement of body weight in malaria has been associated with decreased food intake, disturbed metabolic function and hypoglycemia [31]. Accordingly, prevention of body weight reduction was observed at the middle and higher doses of 80ME treated mice of all models. Besides, body weight loss was less in mice treated with n-butanol fraction followed by chloroform fraction, which could be explained by their relative parasitemia suppression effect. This protective effect is concordant with the findings from some studies [57] and discordant with others [55]. The inconsistency of the results might be due to variation in nutrient content and concentration of appetite suppressing components such as saponins and tannins which were not detected in this study, except tannins in aqueous fraction [58]. The higher effect of crude extract on PCV, rectal temperature and body weight relative to fractions could result mainly due to its, besides to synergistic effect of constituents, higher parasitemia suppression effect [42].

The results of phytochemical screening in both the crude and fractions indicated that the leaf of *O. europaea* is rich in many secondary metabolites including polyphenols, flavonoids, terpenoids, steroids, alkaloids and glycosides. The result is in agreement with previous phytochemical studies done on this plant [59, 60]. There are a number of anti-plasmodial secondary plant metabolites that have shown antimalarial activities belonging to the classes of alkaloids, terpenes, flavonoids, xanthones, anthraquinones, phenolic compounds, sesquiterpenes and other related compounds [61, 62].

Phytochemicals found in 80ME and solvent fractions could have an individual or synergistic effect to exert their antimalarial activity through different proposed mechanisms. Thus, one way of the effect of the plant on *P. berghei* infection may be due to inhibiting the growth and multiplication of the parasite [63]. In view of that, flavonoids have been found to exert their effect by inhibition of the influx of L-glutamine and myoinositol into infected RBCs that are important for parasite growth [64], while steroidal compounds were found to exert their antimalarial activity by changing the membrane of infected RBC and hence block entry of essential nutrients into the RBCs and thereby into the parasite [65]. On the other hand, these phytochemicals could have exerted their action by cytotoxic effect on the parasites [63]. Sesquiterpenes (like artemisinin) and alkaloids (like chloroquine) exert their antimalarial effect by formation of potentially toxic heme-adducts [66, 67]. Similarly, polyphenols may also contribute to the antiplasmodial activity by inhibiting haem polymerization so that the unpolymerized haem is toxic for the parasite [68]. Secondary metabolites may also modulate membrane properties of the erythrocytes, thereby preventing parasite invasion [69]. Besides, phytochemicals like steroids, flavonoids and others may also exert their anti-malarial effects not only by directly affecting the pathogen, but also by indirectly stimulating natural and adaptive defense mechanisms of the host [70, 71].

What is more, studies revealed that the leaf of *O.europaea* possesses potent anti-inflammatory property [56, 60]. The inflammatory condition of malaria is characterized by free radical generation, activation of phospholipase activity resulting in generation of eicosanoids (prostaglandins) and other cytokines such as tumor necrosis factor (TNF), interferon-*γ* (IFN-*γ*) and interlekun-1*β* (IL-1*β*), which up regulate expression of adhesion molecules such as intercellular adhesion molecule-1 (ICAM-1) that is involved in the binding of the parasitized red blood cells to the vascular endothelium [69]. Accordingly, the anti-inflammatory effect of the plant could possibly augment the reduction in overall pathogenic effect of the parasite, in addition to the aforementioned mechanisms, through inhibition of the production and/or release of cytokines.

Consequently, the observed antimalarial activity of the 80ME, in the three models, could be attributed to the presence of secondary metabolites like terpenoids, flavonoids, phenols, alkaloids and other compounds. Likewise, alkaloids, terpenoids, phenols, flavonoids and steroids detected in butanol and chloroform fractions could have contributed to their antimalarial activities, while the low chemo-suppressive effect observed in aqueous fraction might be attributed to a differential distribution of these secondary metabolites in this fraction. Therefore, based on the aforementioned observations it is plausible to assume that the leaf extract of *O. europaea* is a potential antimalarial agent, justifying the claimed use of the plant for malaria control.

## Conclusion

The present study revealed the anti-malarial activity, in all the three models, of the crude extract of the leaves of *O. europaea* and further shown that all the three fractions possessed varying degree of anti-malarial activity, with the n-butanol fraction being the most active fraction in the 4-day suppressive test. The anti-malarial activities of the crude extract as well as fractions could be attributed to the presence of bioactive agents including polyphenols, terpenoids, flavonoids, steroids, alkaloids and glycosides that act individually or together. In addition, the results from the present study suggest that compounds ranging from semi-polar to non-polar are more likely to be responsible for the observed anti-malarial effect.

## Data Availability

All data generated or analyzed during this study and its supplementary information files are included in this published article.

## Abbreviations

80ME: 80% Methanol extract
Ip: Intraperitoneal
MST: Mean survival time
PCV: Packed cell volume
RBC: Red blood cell
SEM: Standard error of the mean
WHO: World Health Organization
